# Testicular immunosenescence: a key player in age-related spermatogenic decline

**DOI:** 10.3389/fcell.2025.1669826

**Published:** 2025-08-13

**Authors:** Ming-Wei Zhan, Mu-Hua Zhou, Bin-Bin Zhao, Xiao-Jie Bao, Yibo Chen, Jingyu Zhu

**Affiliations:** ^1^ Department of Urology, Hangzhou TCM Hospital of Zhejiang Chinese Medical University (Hangzhou Hospital of Traditional Chinese Medicine), Hangzhou, China; ^2^ Department of Urology, Hangzhou Integrative Medicine Hospital Affiliated to Zhejiang Chinese Medical University (Hangzhou Red Cross Hospital), Hangzhou, China

**Keywords:** testicular immunosenescence, aging, spermatogenic dysfunction, immune-metabolic axis, blood-testis barrier (BTB)

## Abstract

As global populations age, testicular aging has become a key contributor to the gradual decline in male fertility, characterized by lower sperm count, poorer sperm quality, and reduced reproductive potential. While the testis is traditionally viewed as an immune-privileged site, growing evidence shows that this immune protection weakens over time—a process now known as testicular immunosenescence. This review provides a comprehensive overview of age-related changes in the testicular immune landscape. These include the depletion of CD4^+^ and CD8^+^ T cells, dysfunction of regulatory T cells (Tregs), abnormal polarization of macrophages, and the breakdown of the blood–testis barrier (BTB). Together, these changes lead to chronic low-grade inflammation and disrupt the delicate environment required for healthy sperm production. In addition, we explore how immune aging is closely linked to metabolic changes, especially within Sertoli and Leydig cells. These intertwined processes form a feedback loop—an “immune–metabolic axis”—that accelerates germ cell death and impairs spermatogenesis. We also discuss emerging treatment strategies, such as anti-inflammatory therapies, mitochondrial support, and NAD^+^ precursor supplementation, which may help preserve testicular function and male fertility with age. By framing testicular immunosenescence as both a driving mechanism and a potential therapeutic target, this review opens up new directions for tackling age-related male reproductive decline.

## 1 Introduction

As the global population ages, the decline in testicular function associated with aging has become an urgent concern in reproductive medicine. Men experience a gradual reduction in spermatogenic capacity with advancing age, marked by decreased sperm count, diminished sperm quality, and impaired fertility (Martins da Silva and Anderson, 2022). This reproductive aging process carries profound implications for men’s overall health, fertility preservation, and the well-being of their offspring.

Traditionally regarded as an “immune-privileged” organ, the testis sustains a unique immunological microenvironment. This immune tolerance is maintained by the blood–testis barrier (BTB), Sertoli cells, and localized immunosuppressive mechanisms, collectively protecting germ cells from autoimmune attack (Zhao et al., 2014). However, recent research reveals that this immune privilege gradually erodes with age. Changes in immune cell populations, increased chronic local inflammation, and impaired immune surveillance collectively define a process now recognized as “testicular immunosenescence” (Kaltsas, 2025; Jiang et al., 2025). Key features of testicular immunosenescence include T cell exhaustion, dysfunction of regulatory T cells (Tregs), abnormal macrophage polarization, and increased permeability of the BTB. These alterations disrupt the fragile immune-spermatogenic balance, promoting germ cell apoptosis and impairing spermatogenesis (Gong et al., 2020; Bhushan et al., 2020). Compared to other organs, the aging trajectory of the testicular immune system remains insufficiently understood—particularly regarding how immune dysregulation intersects with other aging hallmarks such as metabolic imbalance.

This review aims to provide a thorough synthesis of current knowledge on testicular immunosenescence, clarify its central role in age-related spermatogenic decline, and examine its interplay with metabolic reprogramming. By highlighting potential molecular targets and therapeutic avenues, we seek to establish a novel framework for intervention and advance strategies to mitigate male reproductive aging.

## 2 Testicular immunosenescence: features and mechanisms

### 2.1 Age-related changes in the testicular immune system

As the body ages, the testicular immune system undergoes significant changes characterized by increased inflammation, loss of anti-inflammatory cell types, and shifts in immune cell populations. Macrophages, the most abundant immune cells in the testis, show an age-related shift in polarization. Studies in aged mice and humans reveal a tendency for testicular macrophages to polarize toward the pro-inflammatory M1 phenotype, releasing cytokines such as interleukin-6 (IL-6) and TNF-α, which impair the steroidogenic function of Leydig cells in the interstitial tissue (Becker et al., 2018). This reflects a broader pattern of macrophage dysfunction observed during systemic aging. In aged mice, T cells within the testis not only decline in number but also show functional exhaustion. Specifically, testicular CD4^+^ and CD8^+^ T cells from older individuals exhibit increased PD-1 expression and reduced interleukin-2 (IL-2) secretion, classic markers of T cell senescence observed throughout the body (Amodio et al., 2025). At the same time, regulatory Tregs—critical for maintaining immune tolerance—decrease in both quantity and suppressive function. Given the testis’s status as an immune-privileged site, the decline in Tregs likely contributes to chronic local inflammation and damage to spermatogonial cells (Hashimoto et al., 2022). Emerging evidence supports the concept of “inflammaging,” a chronic, low-grade inflammatory state associated with aging. In the testis, this is reflected by elevated pro-inflammatory cytokines and diminished anti-inflammatory immune cell populations. This persistent inflammatory environment accelerates tissue damage and functional decline (Li et al., 2023). The combined effect of immune cell imbalance, impaired immune function, and increased inflammatory mediators sets the stage for T cell exhaustion, BTB breakdown, and disrupted spermatogenesis.

### 2.2 Senescent phenotypes of key immune cells

T cells, Tregs, and macrophages are key players in maintaining immune homeostasis within the testicular microenvironment. Aging drives functional decline in these cells, weakening immune surveillance and promoting chronic inflammation that hastens reproductive decline. T cell exhaustion is a hallmark of immunosenescence. Both CD4^+^ and CD8^+^ T cells in the aging testis adopt an exhausted phenotype, characterized by reduced secretion of cytokines such as IL-2 and IFN-γ. This decline compromises defense against pathogens and disrupts local immune tolerance, potentially allowing germ cells to escape immune surveillance and fostering chronic inflammation (Baessler and Vignali, 2024). Tregs play an essential immunosuppressive role in the testis. With age, both their number and suppressive capacity diminish, evident through decreased FoxP3 expression and reduced secretion of interleukin-10 (IL-10) and TGF-β. This loss likely undermines immune tolerance, exposing spermatogenic cells to immune attack and exacerbating spermatogenic dysfunction (Islam et al., 2024; Yang et al., 2024).

Macrophage polarization also shifts with age, moving from the anti-inflammatory M2 phenotype to the pro-inflammatory M1 state. M1 macrophages secrete cytokines including IL-6, TNF-α, and interleukin-1β (IL-1β), which impair Leydig cell steroidogenesis—leading to lower testosterone production—and sustain chronic local inflammation, further damaging the spermatogenic niche (Chi et al., 2024). Collectively, the senescence and dysfunction of these immune cells contribute to an inflammatory milieu closely tied to aging and impaired testicular function.

### 2.3 Decline of blood–testis barrier integrity and immune imbalance

BTB is fundamental to the testis’s immune privilege, maintained by tight and gap junction proteins that preserve its specialized immune environment. Aging disrupts BTB integrity: the expression of these junctional proteins declines significantly, permitting immune cells and inflammatory factors to infiltrate the testis and upset immune homeostasis (Cui et al., 2025; Wang et al., 2025).

In aged mice, expression of key tight junction proteins such as claudin-11 and occludin is markedly reduced (Martins da Silva and Anderson, 2022; Mruk and Cheng, 2010), increasing BTB permeability. This allows blood-derived cytokines, immune cells, and inflammatory mediators to enter the testicular tissue and interact with germ cells and Sertoli cells. Consequently, macrophages and T cells infiltrate the testis, initiating localized immune activation and sustained inflammation. Such immune cell invasion undermines the immune tolerance that normally protects spermatogenesis. Accumulated cytokines promote germ cell apoptosis and impair sperm production, accelerating reproductive aging (Dutta et al., 2021). Thus, BTB breakdown both facilitates immune infiltration and drives inflammation-induced damage to the spermatogenic environment, marking it as a key mechanism in testicular immunosenescence.

### 2.4 Disruption of the immune environment and its impact on spermatogenesis

Disruption of the testicular immune environment is central to destabilizing the spermatogenic niche. As immunosenescence progresses, declining immune cell function and BTB deterioration permit increased infiltration of pro-inflammatory factors and immune cells. This chronic inflammation not only directly harms spermatogonia but also impairs the supportive roles of Sertoli and Leydig cells, further destabilizing the local microenvironment.

Recent studies highlight lysosomal dysfunction in Sertoli cells during aging and in late-onset hypogonadism (LOH). Aged Sertoli cells exhibit impaired lysosomal acidification, lipid droplet accumulation, and increased pro-inflammatory cytokine secretion, defining a “phago/auto-lysosomal dysregulated” (PALD) phenotype recognized as a hallmark of testicular aging (Tysoe, 2024; Deng et al., 2024). Small molecules that restore lysosomal function have shown promise in reducing lipid accumulation and improving Sertoli cell performance in LOH mouse models. Meanwhile, Leydig cells show reduced steroidogenic capacity, lowering testosterone levels and further compromising spermatogenesis and male reproductive health (Martins da Silva and Anderson, 2022; Matzkin et al., 2021). Altogether, the disrupted immune environment—via direct damage to germ cells and indirect injury to supporting cells—drives the progressive collapse of the spermatogenic niche (Baessler and Vignali, 2024). To illustrate the complex roles of immune cell dysfunction in testicular aging and fertility decline, Table 1 summarizes the key cellular mechanisms underlying testicular immunosenescence.

**TABLE 1:** Mechanistic summary of testicular immunosenescence.

Immune cell type	Age-related functional alterations	Key factors	Phenotypic outcomes	Impact on testicular function	References
Macrophages	Age-associated polarization shift from anti-inflammatory M2 to pro-inflammatory M1 phenotype	IL-6, TNF-α, IL-1β	Increased secretion of pro-inflammatory cytokines; reduced secretion of anti-inflammatory cytokines	Promotes a pro-inflammatory milieu; disrupts homeostasis; impairs testosterone synthesis	(Gong et al., 2020), (Becker et al., 2018), (Chi et al., 2024)
T cells	Functional exhaustion of CD4^+^ and CD8^+^ T cells	PD-1 (upregulated); IL-2, IFN-γ (reduced secretion)	Decline in cytokine production and effector function	Weakened immune surveillance; facilitates chronic inflammation	(Amodio et al., 2025), (Hashimoto et al., 2022), (Baessler and Vignali, 2024)
Treg cells	Decline in cell number and suppressive function	FoxP3 (downregulated); IL-10, TGF-β (reduced secretion)	Impaired immunosuppressive capacity; compromised maintenance of immune tolerance	Breakdown of immune tolerance; increased risk of autoimmune damage	(Hashimoto et al., 2022), (Islam et al., 2024), Yang et al. (2024)
BTB	Structural and functional disruption	Claudin-11, Occludin (downregulated expression)	Reduced expression of tight junction proteins; increased barrier permeability	Loss of immune privilege; infiltration of immune cells and inflammatory mediators	(Wang et al., 2025), (Mruk and Cheng, 2010), (Dutta et al., 2021)
Immune-Metabolic Crosstalk	Immune dysfunction drives metabolic reprogramming, which in turn exacerbates immune imbalance (the “immune–metabolic axis”)	Lactate accumulation (↑ glycolysis); lipid deposition; NAD^+^ depletion; ROS ↑; cGAS–STING and NLRP3 inflammasome activation; SIRT1/NF-κB activation	Enhanced glycolysis in T cells; lipid metabolism disruption in macrophages; mitochondrial dysfunction; oxidative stress; activation of pro-inflammatory signaling pathways	Vicious cycle accelerates global functional decline of testicular cells	(Buck et al., 2017), (West et al., 2015), (Imai and Guarente, 2014), (Zhang et al., 2019), (Aitken and Roman, 2008)

### 2.5 Germ cell apoptosis and spermatogenic impairment

**FIGURE 1:**
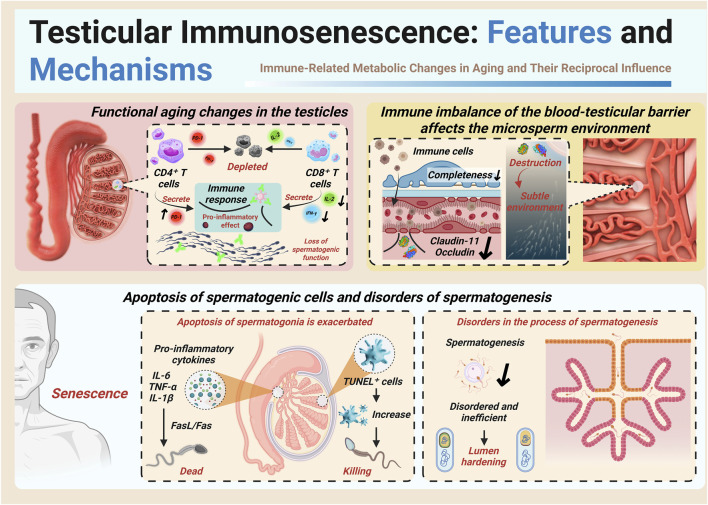
Schematic illustration of testicular immunosenescence and its consequences. The figure summarizes age-associated T cell exhaustion, blood-testis barrier disruption, pro-inflammatory cytokine–induced germ cell apoptosis, and disordered spermatogenesis as key features of testicular aging.

As the testis ages, its once carefully balanced immune environment begins to unravel. The resulting immunosenescence disrupts the delicate cellular ecosystem required for healthy sperm production, ultimately impairing spermatogenesis and accelerating germ cell death, as illustrated in Figure 1. A key feature of this process is chronic, low-grade inflammation, which gradually sets in as part of immune aging. In this inflammatory state, levels of pro-inflammatory cytokines remain persistently elevated in the testicular microenvironment (Matzkin et al., 2021; Dai et al., 2024; Zakariah et al., 2022). These cytokines can activate cell death pathways—such as FasL/Fas and TNF receptor signaling—prompting the premature apoptosis of spermatogonia, the precursors of mature sperm cells. Animal models of experimental autoimmune orchitis (EAO) offer a closer look at these mechanisms. In these models, macrophages infiltrate the testis and release TNF-α, which binds to TNFR1 receptors on spermatogonia, triggering the caspase-8–dependent apoptosis cascade. This process leads to a sharp rise in TUNEL-positive apoptotic cells within the seminiferous tubules. Importantly, neutralizing TNF-α with specific antibodies has been shown to blunt this response, underscoring the destructive potential of pro-inflammatory signaling under chronic immune stress (Matzkin et al., 2021; Zakariah et al., 2022; Ghanem et al., 2011). Aging also brings with it a build-up of reactive oxygen species (ROS), further compounding cellular stress. These free radicals inflict damage on germ cell DNA and cell membranes, pushing already vulnerable cells toward apoptosis. Indeed, studies have documented a marked rise in germ cell death in older testes, along with increased expression of apoptosis-related proteins (Matzkin et al., 2021). At the tissue level, these changes translate into a stark decline in reproductive capacity. Widespread germ cell loss, coupled with damage to the supporting Sertoli cell niche, gives rise to disorganized and inefficient spermatogenesis. Aged testicular tissue often exhibits a characteristic “mosaic” appearance: some seminiferous tubules maintain partial spermatogenic activity, while others are completely depleted of germ cells and show signs of fibrosis (Johnson, 1989). Most aged testes display varying degrees of dysfunction, typically beginning in the later stages of sperm development and gradually extending back to earlier stages. In severe cases, tubules are arrested at the spermatogonial stage—or even emptied entirely, leaving only Sertoli cells behind.

The consequences are clear: reduced production and efficiency of functional sperm (Dong et al., 2022). Human data echo these findings. Even in healthy men, advancing age is associated with declines in semen volume, sperm motility, and the proportion of morphologically normal sperm. Total sperm counts also tend to fall, suggesting that testicular immune aging directly undermines male reproductive potential (Kleshchev et al., 2023). In essence, testicular immunosenescence derails the finely tuned process of spermatogenesis, chiefly by fostering germ cell apoptosis and disrupting the intricate balance required for sperm production.

## 3 Immune-related metabolic changes in aging and their reciprocal influence

### 3.1 The interplay between immunosenescence and metabolic reprogramming

Testicular aging is driven in part by a reciprocal cycle of immune dysfunction and metabolic decline. As men age, immune cells in the testes deteriorate in both number and function. T cells adopt an exhausted phenotype, marked by increased expression of inhibitory receptors such as PD-1 and TIM-3 (Wherry and Kurachi, 2015), alongside a drop in cytokine secretion like IL-2 and IFN-γ (Nikolich-Žugich, 2018). Macrophages shift toward a pro-inflammatory M1 state, secreting high levels of cytokines such as TNF-α and IL-6 (Franceschi et al., 2018). At the same time, regulatory Tregs decrease in both quantity and effectiveness, eroding local immune tolerance (Lages et al., 2008). These immunological shifts in turn reshape cellular metabolism. Exhausted T cells shift from oxidative phosphorylation to glycolysis, resulting in lactic acid accumulation (Buck et al., 2017). M1 macrophages exhibit impaired fatty acid oxidation, leading to lipid build-up (O'Neill and Pearce, 2016), while Treg dysfunction disrupts glutamine metabolism and weakens antioxidant capacity (Angela et al., 2016). Meanwhile, metabolic abnormalities further aggravate immune dysfunction: mitochondrial damage releases mitochondrial DNA, which activates the cGAS–STING pathway and, subsequently, the NLRP3 inflammasome—driving the production of IL-1β (West et al., 2015). Aged-related declines in NAD^+^ weaken SIRT1-mediated inhibition of NF-κB (Imai and Guarente, 2014), while lactate-rich conditions suppress histone deacetylases (HDACs), upregulating pro-inflammatory gene transcription (Zhang et al., 2019). This mutually reinforcing relationship—referred to as the “immune-metabolic axis”—forms a key mechanistic underpinning of chronic testicular inflammation and age-related decline in sperm production (Baessler and Vignali, 2024).

### 3.2 Metabolic decline in leydig cells

Leydig cells, the endocrine engines of the testis responsible for testosterone synthesis, suffer significant metabolic decline with age—especially in mitochondrial function and cellular energy regulation (Zirkin and Papadopoulos, 2018). In a tissue as energy-intensive as the testis, mitochondrial integrity is vital for maintaining homeostasis (Natarajan et al., 2020). Aging disrupts this balance: mitochondrial damage in Leydig cells hampers cholesterol transport via the StAR protein and impairs respiratory Complex I, leading to insufficient substrate supply for testosterone synthesis and decreased ATP production (Miller, 2013). At the same time, increased ROS suppress key enzymes like 3β-HSD (Aitken et al., 2014) and activate TLR4 in macrophages, triggering further TNF-α release and amplifying local inflammation. Germ cells, unable to meet their energy demands during late-stage meiosis through compensatory glycolysis, experience developmental arrest. As the testis’s antioxidant defenses collapse, sperm DNA becomes vulnerable to oxidative fragmentation, further compromising reproductive function (Aitken and Roman, 2008).

Crucially, testosterone deficiency resulting from Leydig cell metabolic decline also undermines systemic Treg activity (Fijak et al., 2011). Meanwhile, lactate accumulation in germ cells fuels further M1 macrophage polarization, deepening the link between metabolic dysfunction and immune aging. In this context, Leydig cell failure is not an isolated event but a central contributor to a broader, self-reinforcing pro-inflammatory cascade involving immune cells such as macrophages and T cells (Hedger, 2011).

### 3.3 The metabolic role of sertoli cells in shaping immune dynamics

Long considered passive “nurse cells” to developing germ cells, Sertoli cells in fact serve as pivotal regulators of both the metabolic and immunological environment of the testis (Xiao et al., 2025). With age, they exhibit classic signs of metabolic dysfunction, including impaired mitochondrial respiration, reduced autophagy, and disrupted energy metabolism (Tysoe, 2024; Zhou et al., 2021). These changes weaken Sertoli cells’ ability to support germ cell development and disrupt their immunoregulatory functions. Aging Sertoli cells produce less energy via oxidative phosphorylation and exhibit diminished autophagic capacity, rendering them less able to maintain the spermatogenic niche (Carrageta et al., 2024). They also begin secreting elevated levels of pro-inflammatory chemokines such as CCL2, which attract immune cells—including macrophages and T cells—into the testicular interstitium (Washburn et al., 2023). Under metabolic stress, Sertoli cells actively promote the polarization of macrophages toward the pro-inflammatory M1 phenotype, further intensifying immune activation.

**FIGURE 2:**
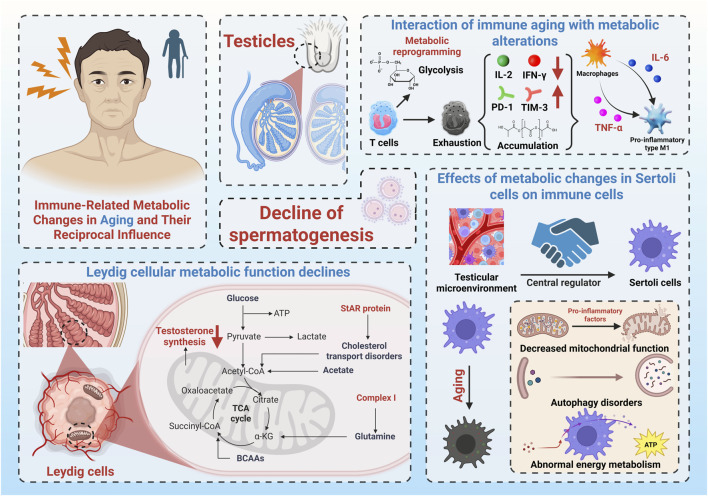
Schematic illustration of testicular immunosenescence and its consequences. The figure summarizes age-associated T-cell exhaustion, pro-inflammatory macrophage polarization, metabolic dysfunction in Leydig and Sertoli cells, and their combined impact on testosterone synthesis and spermatogenesis.

This metabolism-to-immunity feedback loop may underlie the persistent deterioration of the testicular immune environment. In turn, chronic immune activation feeds back negatively on Sertoli cell metabolism through prolonged cytokine exposure. The result is a self-perpetuating cycle—“metabolism, immunity, and damage”—that steadily erodes testicular function. These insights reposition Sertoli cells as active participants, not just passive supporters, in the aging testis. Their central role in regulating immune-metabolic dynamics makes them a promising therapeutic target for interventions aimed at mitigating testicular immunosenescence and preserving male fertility. To provide a visual overview, Figure 2 summarizes the interplay between immune dysfunction, metabolic decline, and the resulting impairment of spermatogenesis during testicular aging. The diagram highlights T-cell exhaustion, pro-inflammatory macrophage polarization, metabolic dysfunction in Leydig and Sertoli cells, and their combined impact on sperm production.

## 4 Therapeutic targets and potential treatments

As scientists learn more about how the immune system in the testes ages, they’re discovering a range of possible treatment options. These new approaches do not just aim to boost metabolism—they also work to calm chronic inflammation, restore immune balance, and repair damaged cells (Meyer and Meyer-Ficca, 2021). Promising strategies include anti-inflammatory drugs, compounds that protect mitochondria (the cell’s energy centers), and supplements that replenish NAD^+^, a molecule essential for cell health (Hacioglu et al., 2021; Wu et al., 2024; Meyer-Ficca et al., 2022)。

### 4.1 Anti-inflammatory therapies

Chronic, low-grade inflammation—often called “inflammaging”—is a hallmark of aging, including in the testes. This ongoing inflammation wears down immune cells and contributes to the decline in testicular function. One key player is IL-6, a pro-inflammatory molecule that becomes overactive with age. High levels of IL-6 have been linked to immune imbalance in the testes, disrupted hormone production, and reduced sperm quality (Bhushan et al., 2020; Zhang et al., 2014; Rival et al., 2006). Drugs like Tocilizumab, which block IL-6, show potential as a way to curb inflammation, rebalance the immune system, and improve reproductive health. In addition to reducing inflammation, IL-6 inhibitors may also help restore testosterone levels (Matzkin et al., 2021; Rival et al., 2006; Potashnik et al., 2005). These therapies could 1 day offer new hope for older men facing fertility issues tied to immune system aging.

### 4.2 Boosting NAD^+^ levels

NAD^+^ is a coenzyme found in every cell, playing a critical role in energy production, DNA repair, and cellular communication. But as we age, NAD^+^ levels drop, which weakens mitochondrial function, reduces the body’s ability to fight oxidative stress, and accelerates immune aging. One solution? Supplementing with NAD^+^ precursors—molecules that help the body make more NAD^+^ (Wu et al., 2024; Meyer-Ficca et al., 2022). Research shows that these supplements can jump-start important cellular processes: activating protective enzymes like SIRT1, improving energy metabolism, reducing inflammation, and strengthening immune function (Braidy and Liu, 2020). One standout compound, NMN (nicotinamide mononucleotide), has shown impressive results in improving sperm quality in pigs. It raises NAD^+^ levels, lowers harmful ROS, boosts ATP (the cell’s energy currency), and supports healthy mitochondria—all without interfering with other energy pathways. In fertility trials, sperm treated with NMN led to more healthy piglets and fewer stillbirths (Zhang et al., 2025). Restoring NAD^+^ doesn’t just help metabolism—it can also rejuvenate aging immune cells. As a result, NAD^+^-boosting compounds may offer a new path forward in treating testicular aging and improving fertility in older men (Chini et al., 2020; Rahman et al., 2024).

### 4.3 Protecting the mitochondria

Mitochondria are the powerhouses of the cell—and when they falter, so does testicular function. As men age, their mitochondria become less efficient, which not only disrupts metabolism but also fuels inflammation and damages sperm-producing cells (Ham et al., 2024). One potential solution comes from Elamipretide, a mitochondrial-targeted drug that helps restore energy production, reduce oxidative stress, and improve overall cell health (Zhao et al., 2019). In studies on rooster sperm, adding Elamipretide during the freezing and thawing process significantly boosted sperm vitality, membrane integrity, and mitochondrial function, while lowering oxidative damage and cell death. However, researchers caution that too high a dose may have toxic effects (Najafi et al., 2025). By strengthening antioxidant defenses and supporting energy metabolism, Elamipretide and similar drugs could become valuable tools in fighting age-related infertility—especially when poor mitochondrial function is to blame.

### 4.4 New tools and future directions

**FIGURE 3:**
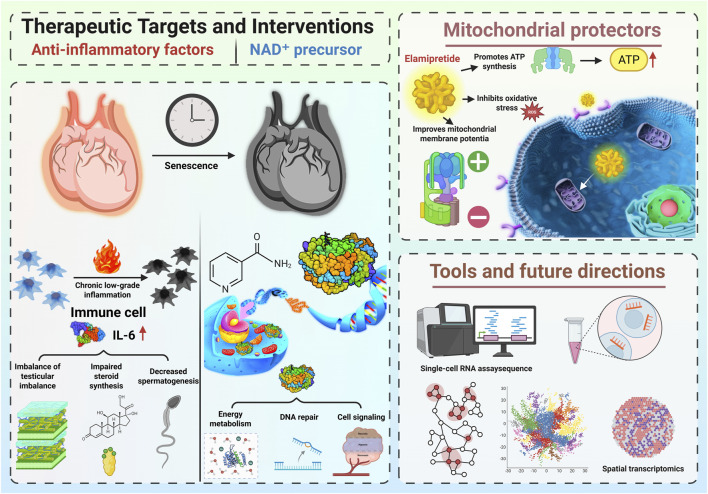
Therapeutic strategies and emerging technologies targeting testicular aging. The figure highlights anti-inflammatory therapies (e.g., IL-6 inhibitors), NAD + precursors for energy metabolism and DNA repair, mitochondrial protectors such as Elamipretide, and advanced research tools like single-cell RNA sequencing and spatial transcriptomics.

Thanks to cutting-edge technologies like single-cell RNA sequencing and spatial transcriptomics, researchers can now study aging at an unprecedented level of detail. These tools reveal how different testicular cell types interact and change over time. Single-cell analysis shows how immune cells shift metabolically with age, while spatial mapping pinpoints where these changes occur within the tissue (Chi et al., 2024; Cui et al., 2025). Looking ahead, gene editing technologies such as CRISPR-Cas9 could allow scientists to precisely tweak genes that control immune responses, offering new ways to delay or even reverse testicular aging (Li et al., 2015). These innovations not only make it easier to build accurate disease models but could eventually lead to personalized treatments for male infertility. Figure 3 illustrates the therapeutic strategies and future research directions aimed at combating testicular immunosenescence. It includes anti-inflammatory therapies targeting IL-6, NAD^+^ precursors for energy metabolism and DNA repair, mitochondrial protectors like Elamipretide, and advanced tools such as single-cell RNA sequencing and spatial transcriptomics.

## 5 Conclusion and perspectives

This review highlights how immune system aging and metabolic dysfunction work together to drive the decline of male reproductive health. It introduces the idea of an “immune–metabolic axis”—a feedback loop where inflammation weakens the BTB, rewires immune cell metabolism, and disrupts sperm and testosterone production. At the same time, age-related problems like poor glucose handling, sluggish mitochondria, and imbalanced fat metabolism make these immune issues even worse. In response, multi-pronged treatment strategies—such as anti-inflammatories, mitochondrial boosters, and NAD^+^ supplements—show real promise. To fully understand and refine these therapies, future research should combine advanced tools like spatial omics, CRISPR gene editing, and lab-grown organoid models. Large-scale clinical trials are also needed to test treatment regimens and identify biomarkers that predict success.

Ultimately, the concept of the immune–metabolic axis opens up new ways to understand and treat testicular aging. With continued research, it could offer a roadmap for protecting fertility and reproductive health in aging men.
