# Correction: Testicular immunosenescence: a key player in age-related spermatogenic decline

**DOI:** 10.3389/fcell.2025.1687852

**Published:** 2025-09-03

**Authors:** Ming-Wei Zhan, Mu-Hua Zhou, Bin-Bin Zhao, Xiao-Jie Bao, Yibo Chen, Jingyu Zhu

**Affiliations:** ^1^ Department of Urology, Hangzhou TCM Hospital of Zhejiang Chinese Medical University (Hangzhou Hospital of Traditional Chinese Medicine), Hangzhou, China; ^2^ Department of Urology, Hangzhou Integrative Medicine Hospital Affiliated to Zhejiang Chinese Medical University (Hangzhou Red Cross Hospital), Hangzhou, China

**Keywords:** testicular immunosenescence, aging, spermatogenic dysfunction, immune-metabolic axis, blood-testis barrier (BTB)

Affiliation “^1^Department of Urology, Hangzhou TCM Hospital of Zhejiang Chinese Medical University (Hangzhou Hospital of Traditional Chinese Medicine), Hangzhou, China” was omitted for author Jingyu Zhu. This affiliation has now been added for author Jingyu Zhu, and the updated affiliations for this author are “^1,2^”.

The original version of this article has been updated.

